# The effect of changing constituents on tensile mechanical properties of HfNbTaTiZr high entropy alloy: A molecular dynamics study

**DOI:** 10.1016/j.heliyon.2024.e38350

**Published:** 2024-09-24

**Authors:** Wasif Abu Dujana, Sazzad Ahmad, Md. Nazmul Haque Noman, Mohammad Humaun Kabir

**Affiliations:** a Department of Materials and Metallurgical Engineering, Chittagong University of Engineering & Technology, Chattogram, 4349, Bangladesh; b Department of Materials Science and Engineering, Khulna University of Engineering & Technology, Khulna, 9201, Bangladesh

**Keywords:** High entropy alloys, Mechanical properties, Molecular dynamics, Alloy design, Surface-contour plot

## Abstract

The recent trend of high-entropy alloys (HEAs) was studied extensively for their promising mechanical properties, but individual constituents' effects have remained unexplored. In this work, the effects of changing the percentage of elements of HfNbTaTiZr-HEA on the mechanical properties were analyzed during uniaxial tension using molecular dynamics simulation. The tensile strength and modulus of elastic properties of the samples were analyzed. It was found that adding Nb or Ta up to 10 % (*i.e.* Nb10/Ta10) in the high entropy alloys increased the ultimate tensile strength (UTS) from 2.9 GPa in the base alloy to 3.8/3.9 GPa (Nb10/Ta10) respectively, but further increment of these elements to 30 % resulted in a downgrade of UTS to 2.7 GPa. Similarly, the modulus of elasticity increased from 117.7 (±3) GPa in the base alloy to 137.7/129 (±3) GPa (Nb10/Ta10) respectively, but fell to 112–115 GPa upon further increment. The initial increase in strength could be due to the solid solution strengthening mechanism. However, further increases in these elements might hinder the development of a homogeneous solid solution because of differences in atomic size and crystal structure, which could ultimately reduce the alloy's strength. However, the effect of Ti and Zr follows an opposite trend as compared to Nb and Ta. Furthermore, the optimum composition of HEAs alloys was analyzed using a surface-contour plot and suggests minimizing the inclusion of Ta for maximizing the UTS, E, and %Elongation. Also, the high-temperature behavior of the optimized HEA's alloy was analyzed which showed a deterioration in properties at elevated temperature. The fracture evolution of the samples showed cup and cone-type fractures propagating under strain, the linear thermal expansion coefficient of HfNbTaTiZr-HEA was also calculated and found closer to the literature value.

## Highlights

• Molecular Dynamics simulation was employed to obtain thermo-mechanical properties of HfNbTaTiZr HEA.
• Among 13 alloy models, Nb10 and Ta10 showed a superior combination of strength and Modulus of elasticity, whereas Ta10 showed a superior property in terms of %Elongation.
• The alloy with no Titanium has better thermo-mechanical properties at high temperature.
• The two optimized alloys have better TEC than HfNbTaTiZr.
• Molecular dynamics reveals cup and cone features at the fractured surfaces.

## 1 Introduction

The high-entropy alloy (HEA) has been gaining more attention lately due to its exceptional properties such as high strength, significant hardness, and excellent wear resistance [1,2]. Apart from conventional alloys, a HEA consists of a minimum of four or five constituent elements, each present in a molar ratio ranging from 5 % to 35 % [3]. To construct HEAs, a wide range of materials have been used in recent times. Despite the vast number of possible combinations of four or five constituent elements, it is possible to identify five primary groups of metallic HEAs [4], which are FCC-Cantor Alloys, BCC-Refractory Alloy, HCP-HEAs, Light Elements Alloy-HEAs, and the HEAs that possess valuable functional properties [5]. Among these, the BCC refractory HEAs are distinguished from other materials by their outstanding high-temperature strength, oxidation resistance, and corrosion resistance properties has gained significant attention. These alloys primarily consist of refractory elements such as tungsten, tantalum, molybdenum, zirconium, hafnium, vanadium, and niobium, each present in the range of 5 %–35 % [6].

The current research focused on the refractory HEA, which is constructed by the elements from the I, V, and VI groups of the periodic table [7]. The various mechanical properties of HfNbTaTiZr system has been experimentally studied by the researchers. The tensile creep behavior of HfNbTaTiZr refractory HEA has been demonstrated by Liu et al. [8] at 1250 °C and suggested that the creep rate of the alloy was controlled mainly by Ta element. Another study by Zherebtsove et al. [9] examined the microstructure and mechanical properties of HfNbTaTiZr-HEA during cold rolling. While increasing the thickness strain to 15 % the microhardness was increased and came to a plain stage at the interval of 15–40 % strain, afterword the microhardness again increased with the thickness strain. The elastic moduli at higher temperatures ranging from 293 K to 1100 K were also explored by Laplanche et al. [10]. Microhardness was strongly increased after aging the HfNbTaTiZr-HEA at 600 °C and a second HCP phase particles were formed in the BCC matrix after the process of annealing at 600 °C and 800 °C [11]. The HfNbTaTiZr-HEA is being used in dual-phase alloys [12] and reinforced high entropy composites [13,14]. Dissimilarity in thermal expansion coefficient can lead to thermal stresses during solidification, which potentially might affect the composite strength [15]. Therefore, this study also aims to understand the thermal expansion coefficient (TEC) and elastic moduli at specific temperatures.

Though it is difficult to find hafnium (Hf) that is pure due to its high reactivity in the ambient atmosphere [4], also as both hafnium and tantalum are high price elements [16,17], it is difficult to experimentally study the effect of all the elements on mechanical properties of HfNbTaTiZr-HEA. Adding to that, a more sophisticated and costlier experimental setup is necessary for high temperature inspections. Moreover, there is lack of studies that show the effect of changing the ratio of all five constituent elements of HfNbTaTiZr-HEA on the mechanical properties, especially the stress-strain behavior. Hence, the current research focused on molecular dynamics (MD) simulation to study the effect of changing composition on stress-strain behavior in the present study. The current study shows the effect of changing the Nb, Ta, Ti, and Zr elements from 0 to 30 % on stress-strain behavior of the HfNbTaTiZr-HEA and also gives an insight into the fracture that occurred during the uniaxial tensile test. Overall, how the changes in the atomic level interaction and crystal structure affect the macroscopic properties of HEA are investigated. This level of detail is difficult to achieve experimentally making our findings particularly valuable. Also, the understanding can contribute to the design of new HEA with more tailored properties for various engineering applications such as nuclear or aerospace industries. TEC was also investigated for the equimolar alloy and for the optimized alloys which are discussed in the later section of this article.

## 2 Computational methods

In this study, molecular dynamics simulations (MD) have been applied with the aid of Large-Scale Atomic/Molecular Massively Parallel Simulator (LAMMPS) code [18] and the visualization and post-processing were done by the software OVITO [17]. We have utilized the Modified Embedded Atom Method (MEAM) potential function for HfNbTaTiZr developed by Huang et al. [19], which has been validated by their previous work. The overall potential energy for this model is expressed by equation (1) [20],

(1) $$E=\sum_{i}F_{i}+\frac{1}{2}\sum_{j\neq i}S_{ij}\phi_{ij}$$

Where, $F_{i}$ is the embedding energy, $S_{ij}$ is the screening function, and $\phi_{ij}$ is the pair energy between two atoms. MEAM has been chosen for this study because the model has better performance in terms of multi-component alloys where a range of complex bindings exists. Also, the model is aligned with results found experimentally which suggests the validity of the used model [19]. A BCC unit cell of lattice constant equal to 3.403 Å [21] of HfNbTaTiZr-HEA cubic crystal was taken and the simulation box was strained in the z-direction for calculating their mechanical properties with a strain rate of 1 × 10^8^ s^−1^. We have applied the periodic boundary conditions (p p p) in all three directions of the simulation cell. The atoms can interact across the boundary allowing them to exit from one end of the simulation box and re-enter from the opposite end. This boundary condition can avoid the effect of boundaries that are generated for the finite size of the particles. A total number of 13 different models of HfNbTaTiZr-HEA have been created with varying percentages of elements, shown in Table 1. During the variation of the composition, ‘Hf’ was chosen as a base element, therefore the change of composition was merged to Hf % only. The other four elements (i.e. Nb, Ta, Ti, and Zr) with four compositions i.e., 0 %, 10 %, 20 %, and 30 % create a total 16 (4 × 4) HEA-configurations where only 13 are unique. The composition range was chosen based on the available data from the literature [6]. This will allow us to observe systematically how small changes in concentration affect the properties. Each model has the dimension 20a × 20a × 50a, containing 40,000 atoms in total. The system temperature was controlled at 300 K with isobaric-isothermal (NPT) ensemble. The time step was set to 1fs using the integration of motion equations using the Varlet leapfrog method. The minimization of total energy and interatomic forces among the atoms was done to ensure the equilibrium state using the LAMMPS environment. Before applying the tensile load, the system was relaxed for 30ps to an equilibrium state. The isothermal-isobaric ensemble was used to equilibrate the pressure and to adjust the temperature as prescribed earlier.

**Table 1:** The percentage of elements for the constructed models of HfNbTaTiZr -HEA.

HEA- ID	%Hf	%Nb	%Ta	%Ti	%Zr
Nb 0	40	0	20	20	20
Nb 10	30	10	20	20	20
Nb 20^a^	20	20	20	20	20
Nb 30	10	30	20	20	20
Ta 0	40	20	0	20	20
Ta 10	30	20	10	20	20
Ta 20^a^	20	20	20	20	20
Ta 30	10	20	30	20	20
Ti 0	40	20	20	0	20
Ti 10	30	20	20	10	20
Ti 20^a^	20	20	20	20	20
Ti 30	10	20	20	30	20
Zr 0	40	20	20	20	0
Zr 10	30	20	20	20	10
Zr 20^a^	20	20	20	20	20
Zr 30	10	20	20	20	30

a Nb 20/Ta 20/Ti 20/Zr 20 is the equimolar, Hf_0.2_Nb_0.2_Ta_0.2_Ti_0.2_Zr_0.2_- HEA composition.

## 3 Results and discussion

HEA stands out among other materials in many engineering fields due to its strength and modulus of elasticity being unparalleled, making it a top choice for various applications, including aerospace, materials for resistance to damage, and macro to micro-scale tool materials. As such, there has been a multitude of experimental studies conducted to comprehend the deformation behaviors of different HEA [22,23]. The Ultimate Tensile Strength, Modulus of Elasticity, and %Elongation of different combinations of HfNbTaTiZr-HEA constituents are discussed in this section. In addition, the effects of external perturbations such as temperature on the mechanical properties of the alloy were investigated along with the evaluation of TEC.

### 3.1 Individual elastic behavior of the single elements

The ideal stress-strain behavior of the individual constituent elements was tabulated for comparison purposes. Table 2 gives the comparison of Elastic Moduli of individual HEA elements where ‘Ta’ has exhibited the value of C_11_, and Young's Modulus among the other constituents of HfNbTaTiZr-HEA elements of this group. The BCC crystal of ‘Ta’ mainly contributes to its high strength and modulus by limiting the pathways for movement of dislocations that require high stress to deform plastically. On the contrary, ‘Zr’ exhibits the lowest elastic Moduli as well as the C_11_ value, when compared to BCC and FCC structures, Zr's hexagonal close-packed (HCP) structure has fewer slip systems. The prismatic planes make plastic deformation more easier in ‘Zr’ that lower the Critical Resolved Shear Stress (CRSS) and also lowers tensile strength and ductility [24]. Moreover, the experimental results of the different groups of researchers also confirmed that the ‘Ta’ exhibits the highest Young's Modulus when compared to other constituent elements of the HfNbTaTiZr-HEA. The Comparative data of the theoretical and the experimental findings are shown in Table 2.

**Table 2:** Summarization of individual mechanical properties of HfNbTaTiZr-HEA constituents.

Elements	Crystal Structure	Atomic Radius (pm)	C_11_ (GPa)	Young's Modulus (GPa)	Bulk Modulus (GPa)
Hf	HCP	225	183.9^a^ [26]	115^b^ [27]	108.8^a^ [26]
			190.1^b^ [28]		118^a^ [29]
			184.4^a^ [29]		109^b^ [30]
Nb	FCC	207	250^a^ [31]	103.8^b^ [32]	173^a^ [31]
Ta	BCC	220	262.6^a^ [33]	186^b^ [34]	
				181.9^a^ [33]	
Ti	HCP	147	170.04^a^ [35]	116^a^ [36]	109.9^b^ [37]
			176.1^b^ [38]	130.62^b^ [37]	
Zr	HCP	160	155^a^ [39]	94.5^b^ [40]	
				114^a^ [41]	

a Simulated Result.

b Experimental Result.

From Table 2 we can also see that there are some extents of variation between the experimental and theoretical Modulus. The reasons for the variation of experimental results and the simulated results depend on many factors. Fabrication methods are one of them whereas post-processing heat treatment, strain rate, and defect density are the other contributing factors for lower experimental value than theoretical one. For instance, variations in heat treatment process parameters can induce microstructural alterations thereby affecting the mechanical properties. Improper annealing or quenching may add residual stresses or may alter the phase composition in the structure [25]. Consequently, this affects the structure's response to external perturbations such as loading environments. Various mechanical responses can be exhibited due to strain rate sensitivity. The loading rates and temperature in real conditions can affect the phenomenon resulting in overestimation or underestimation of the material's properties. Defect densities are another factor for the lower experimental values than theoretical ones.

### 3.2 Effects of elemental variations on stress-strain behavior

**Fig. 1:**
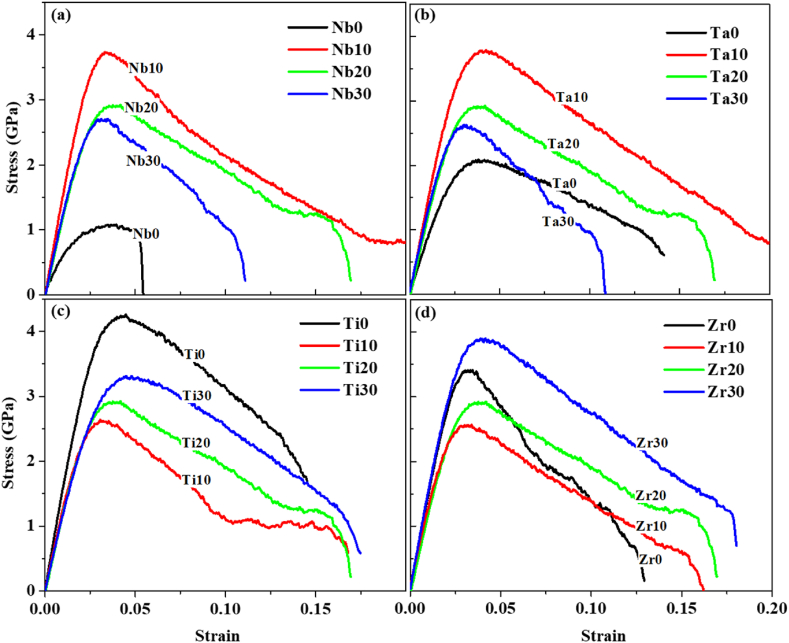
Stress vs Strain Curve of different compositional combinations of HfNbTaTiZr-HEA material: (a) Nb, (b) Ta, (c) Ti, and (d) Zr variations.

The influence of elements on the mechanical properties of the HEAs is observed by varying the compositions of the alloy, where the strain rate was maintained at 1 × 10^8^ s^−1^ for 300 K of temperature. The stress-strain curves for all the samples are plotted in Fig. 1.

Fig. 1 illustrates the stress-strain curves for four different compositional combinations of HfNbTaTiZr-HEA material, which can be utilized to find out the mechanical properties. There are three different zones in the stress-strain plot, such as linear elastic zone, nonlinear elastic zone, and nonlinear plastic zone. The linear portion of the curves, where the stress-strain behavior is proportional, can be demonstrated by Hooke's law. The calculated different mechanical properties are tabulated in Table 3**.**

**Table 3:** Mechanical Properties of different chemical compositions of HfNbTaTiZr-HEA.

HEA-ID	UTS (GPa)	Modulus of Elasticity (GPa) b	Elongation (%)
Nb 0	1.1	58.3	5.42
Nb 10	3.8	137.7	19.35
Nb 20^a^	2.9	117.7	17.00
Nb 30	2.7	112.2	11.17
Ta 0	2.1	78.0	14.58
Ta 10	3.9	129.1	25.51
Ta 20^a^	2.9	117.7	17.00
Ta 30	2.7	115.0	10.85
Ti 0	4.3	137.3	14.54
Ti 10	2.7	115.0	16.53
Ti 20^a^	2.9	117.7	17.00
Ti 30	3.4	118.2	17.53
Zr 0	3.5	133.6	12.98
Zr 10	2.6	114.6	16.56
Zr 20^a^	2.9	117.7	17.00
Zr 30	3.9	124.0	18.10

a Nb20/Ta20/Ti20/Zr20 is the equimolar HEA composition.

b The error analysis for the modulus of elasticity is ±3 GPa.

#### 3.2.1 Effects on ultimate tensile strength (UTS)

**Fig. 2:**
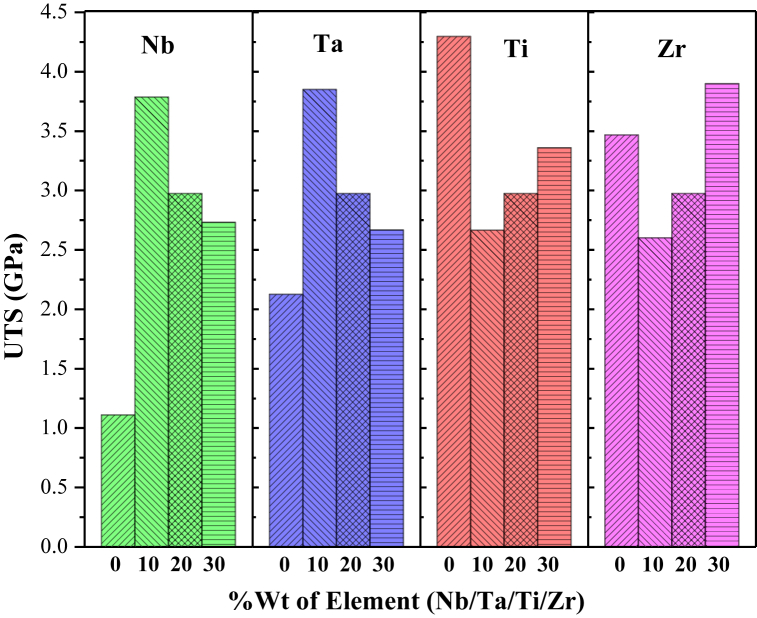
Material composition effect on ultimate tensile strength.

The effect of changing constituents and their respective composition were studied to explore their effects on UTS. Fig. 2 demonstrates how the UTS property of the HfNbTaTiZr-HEA changes when Nb, Ta, Ti, and Zr percentages are changed individually.

The tensile strength of the material was 1.1 GPa for Nb0 meaning there are only four elements in the alloy and increased to 3.8 GPa when Nb is added to HEA of 10 % *i.e.* simply by adding Nb can make the alloy stronger. The tensile strength is raised here as a result of the ‘Nb’ addition is mainly of solid solution strengthening effect [42]. A small amount of Nb can disrupt the regular arrangement of atoms in the host material and impede the mobility of the dislocations and cause plastic deformation. In other words, when a high-strength element Nb (*i.e.* 12.08 GPa) is added to a lower-strength Hf_0.4_Nb_0.0_Ta_0.2_Ti_0.2_Zr_0.2_-HEA alloy (*i.e.*, 1.1 GPa), it increases the strength initially. However, the alloy's strength falls as the ‘Nb’ content increases, specifically for the cases of Nb20 and Nb30. The mismatch between ‘Nb’ and the base metal also grows as ‘Nb’ content rises due to the difference in atomic size and crystal structure. Additionally, at a certain point, increasing the ‘Nb’ level in the alloy might prevent the development of a homogeneous solid solution, lowering the alloy's strength.

The composition change of ‘Ta’ exhibits similar phenomena. The alloy's tensile strength is 2.1 GPa when ‘Ta’ is absent in the HEA and simply by adding ‘Ta’ (*i.e.,* Ta10), the strength increases to 3.9 GPa and further increase leads to gradual falls in the tensile strength for Ta20 and Ta30. The ‘Nb’ and ‘Ta’ both have a cubic structure (FCC and BCC respectively) and have a closer atomic radius (207 p.m. and 220 p.m.), as presented in Table 2, therefore they would have followed a similar trend in UTS properties during composition changes.

On the other hand, the addition of ‘Ti’ follows an opposite trend compared to ‘Nb’ and ‘Ta’. When ‘Ti’ is absent, the Hf_0.4_Nb_0.2_Ta_0.2_Ti_0.0_Zr_0.2_ -HEA alloy at its peak value of 4.3 GPa and suddenly dropped to 2.7 GPa due to the addition of 10 % of the referred element. Furthermore, it was observed that as the Ti % rises, so does the material's strength. It might be due to the initial addition of ‘Ti’ atoms occupying the substitutional position within the crystal lattice to form a solid solution. This can initially disrupt the orderly arrangement of atoms, causing lattice strain and potentially creating new defects. These defects can act as sites for crack initiation and propagation, leading to a decrease in strength. However, with the increasing addition of the element, the surrounding atoms can rearrange and accommodate the strain, leading to a stronger and more ordered structure. This results in an increased strength compared to the initial decrease. The addition of ‘Zr’ shows very similar effects on the HEA alloy as described for the ‘Ti’ element. It can be seen from Table 2 that the ‘Ti’ and ‘Zr’ both have the HCP crystal structure and closer atomic size difference (147 p.m. and 160 p.m. respectively) therefore it is expected they have similar effects on the properties.

**Fig. 3:**
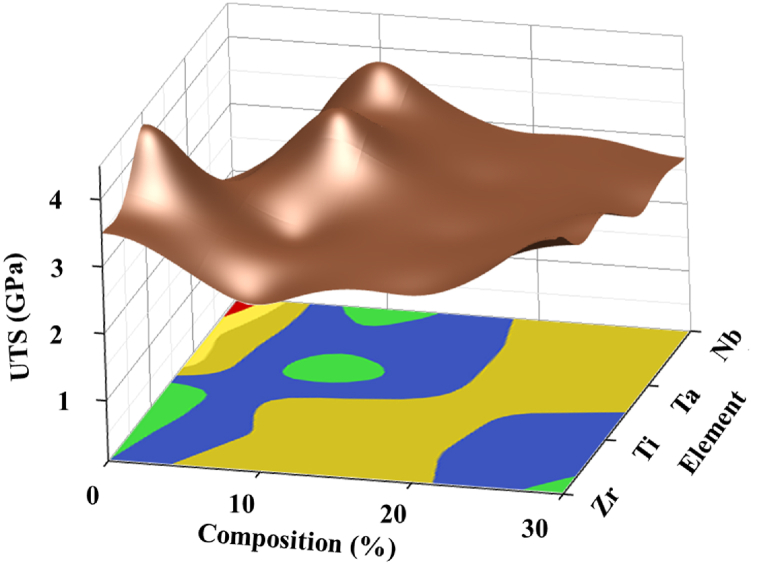
Surface-Contour plot showing the effects of HEA constituent and composition on Ultimate Tensile Strength property.

To investigate the optimum UTS property for the best combination of constituent elements, their respective composition was analyzed using a surface and contour plot. Fig. 3 illustrates that among the four constituent elements (Nb/Ta/Ti/Zr) of HEA alloy: Nb10, Ta10, and Ti0 exhibit the maximum tensile strengths and make them the most suitable for high-strength applications. The contour plot at the bottom confirms the same by indicating the green region for the maximum strength corresponding to Nb10, Ta10, and Ti0 composition.

#### 3.2.2 Effects on modulus of elasticity (E)

**Fig. 4:**
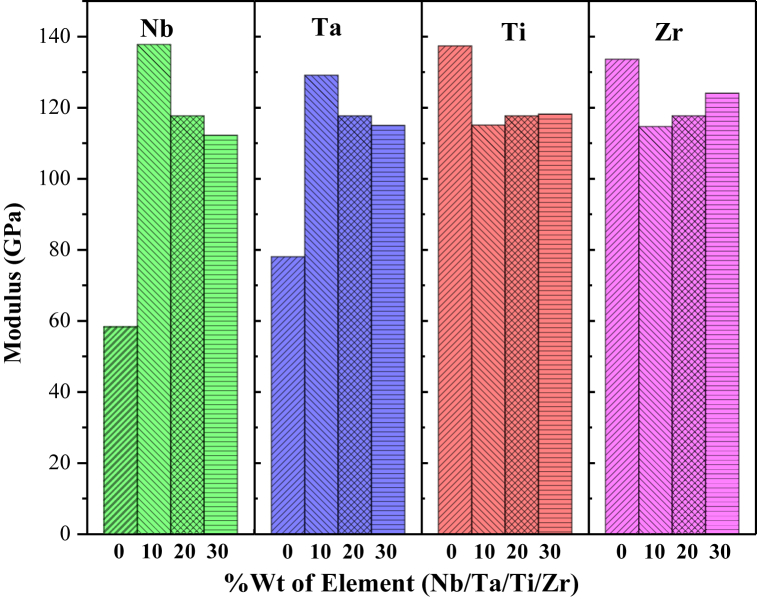
Material composition effect on modulus of elasticity.

The effects of material composition on modulus of elasticity are depicted in Fig. 4 and the results are identical with the composition variation on UTS. The highest observed value for the elastic modulus was recorded for the 'Nb' variation, specifically at Nb10, reaching 129.1 GPa. Similarly, while varying the ‘Ta’, it was found 137.7 GPa at Ta10. The reasons might be the same as discussed earlier for the UTS property.

However, the addition of ‘Ti’ in HfNbTaTiZr-HEA decreases the modulus value from 137.3 GPa to 115.0 GPa for Ti0 and Ti10, respectively. Further addition of ‘Ti’ to the HfNbTaTiZr-HEA would increase the elastic modulus to 118 GPa at Ti = 30 %. A similar effect was observed for ‘Zr’ addition. The value of modulus of elasticity obtained for the HEA with four other constituent elements except ‘Zr’ was 133.6 GPa. Further addition of ‘Zr’ follows the lowest value (114 GPa) to a progressive increasing pattern but slowly similar to ‘Ti’. In other words, when an HCP element (*i.e.,* Ti/Zr) was introduced to a BCC-HEA alloy the modulus was decreased initially and remained almost unchanged for a higher percentage.

**Fig. 5:**
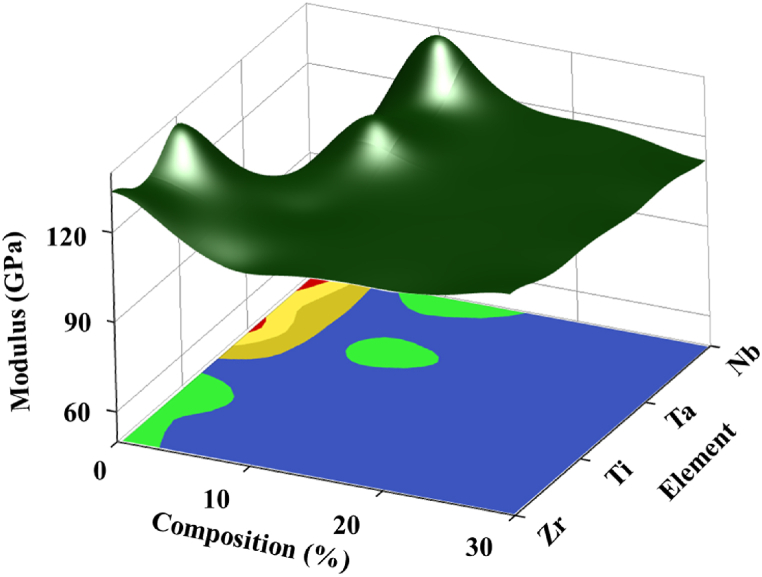
Surface-Contour plot showing the effects of HEA constituent and composition on Modulus of Elasticity property.

Fig. 5 illustrates the combined effects of changing HEA constituents and compositions on Elastic Modulus through a surface-contour plot. It was found that ‘Nb’ at 10 % exhibits the maximum modulus of 137.7 GPa among all the combinations. Additionally, ‘Ta’ at 10 % and ‘Ti’ at 0 % could be an optimum choice for HEA materials when requiring a higher modulus, indicated by a green area in the contour plot.

#### 3.2.3 Effects on elongation

**Fig. 6:**
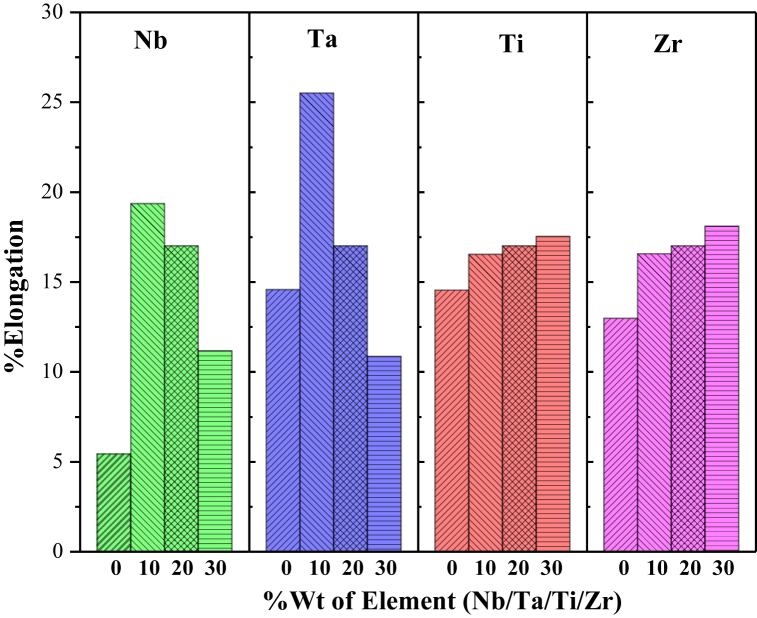
Material composition effect on %Elongation.

The effects of changing elements and their respective composition are illustrated in Fig. 6 where ‘Nb’ and ‘Ta’ follow a similar trend and ‘Ti’ and ‘Zr’ follow an opposite pattern.

Fig. 6 demonstrates that the addition of 'Nb' in a small percentage results in a notable alteration in elongation. However, subsequent increments of this element may diminish elongation properties due to pronounced strengthening effects. Similarly, the addition of ‘Ta’ element increases the sample elongation ranges from 10 % to 25 %. Furthermore, ‘Ti’ and ‘Zr’ presence in the sample, pushes the elongation upward between 14 % and 18 % but very slowly as compared to the other two elements. The elongation of the material depends on the composition of the material, which affects the dislocation behavior. Atomic mismatch and the strengthening of solid solutions are further causing this.

**Fig. 7:**
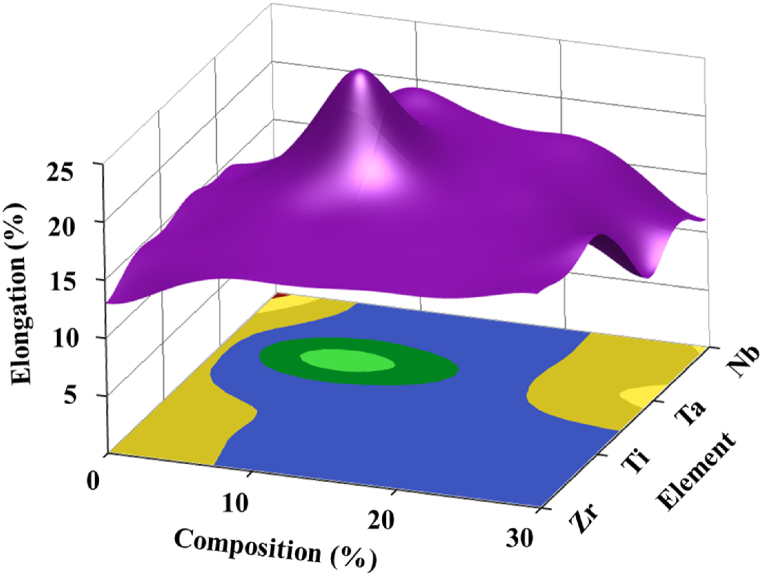
Surface-Contour plot showing the effects of HEA constituent and %phase on %Elongation.

Fig. 7 illustrates the surface-contour plot of the HEA constituent and composition effects on the elongation property. It signifies that the percentage of elongation of the HEA samples shows a maximum at Ta10, indicated by the green region in Fig. 7. Therefore, this composition could be a better choice to optimize the ductility of the HfNbTaTiZr-HEA.

### 3.3 Temperature effects of on optimized HEA compositions

From the above discussion, it was concluded that Nb10, Ta10, and Ti0 offer the best combination of UTS, Modulus of Elasticity, and %Elongation among all other compositions. Therefore, the temperatures of 300–1200 K at 300 K intervals are used to understand the effects of temperature. The purpose of this test is to comprehend the high-temperature behavior of these alloys while used as refractory or thermal shield material.

**Fig. 8:**
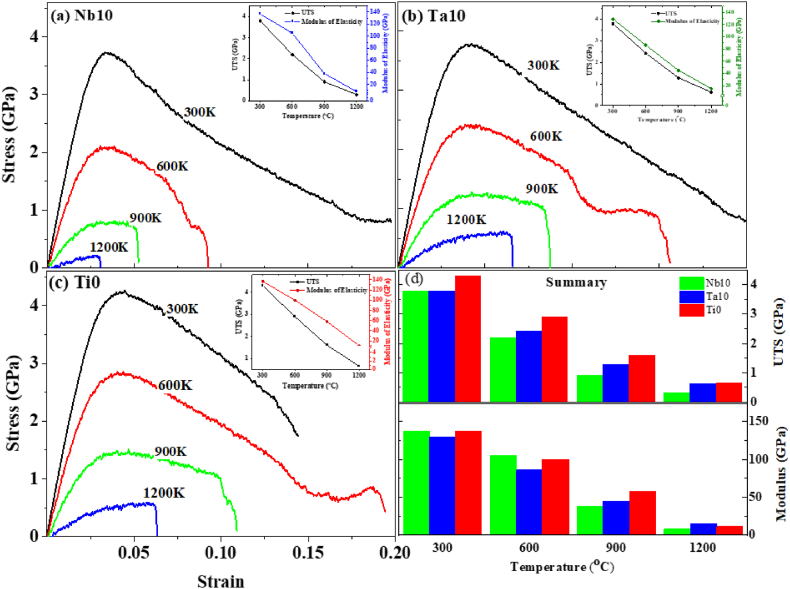
Temperature effects on Stress vs Strain behavior of optimized composition in HEA.

The UTS and modulus of elasticity of Nb10-HEA (i.e. Hf_0.30_Nb_0.10_Ta_0.20_Ti_0.20_Zr_0.20_) alloys are 3.7 GPa and 137.7 GPa at room temperature, respectively (see in Fig. 8(a)). At a high-temperature exposure *e.g.* 1200 K, it shows the UTS value of 300 MPa and 8.35 GPa for modulus of elasticity. The values of UTS and modulus of elasticity steadily decrease as the temperature rises. This is brought on by thermal vibrations within the lattice. An increase in thermal vibration promotes the dislocation's migration thus decreasing the strength of the materials [43].

Similar effects were also observed for another two optimized HEA compositions (*i.e.* Ta10 and Ti0). The strength and modulus were decreased gradually with the increasing temperature, as shown in Fig. 8(b) and (c). Fig. 8(d) illustrates the overall effects of temperature on the stress-strain behavior of the aforementioned HEA compositions. The overall effects of temperature on the variation of composition did not significantly alter the high-temperature properties. However, Ti0 showed comparatively better strength and modulus of elasticity than the other two compositions.

#### 3.3.1 Linear thermal expansion coefficient

The linear thermal expansion coefficient of the optimized HEA composition i.e. Nb10, Ta10, and Ti0 was investigated where the structure was heated from 300 K to 1200 K with a heating rate of 0.02 K/fs. The increase in length was documented to calculate the thermal expansion coefficient using the following equation [44]:

(2) $$\alpha =\frac{1}{L_{o}}\;\frac{dL}{dT}$$

*L*_*o*_ = Initial Length of the structure.

*dL/dT* = Rate of change of length with respect to change in temperature

*α* = Thermal Expansion Coefficient.

HEAs are increasingly valued for their thermal properties, particularly the thermal expansion coefficient (TEC), which is pivotal in ensuring operational integrity across various demanding applications. The TEC is crucial for maintaining dimensional stability and managing internal stresses within materials subject to temperature fluctuations. For instance, in the aerospace and automotive sectors, HEAs are utilized in engine components where matching the TEC with adjacent materials minimizes stress and enhances durability. Similarly, HEAs also play a significant role in thermal barrier coatings by aligning their expansion characteristics with those of the substrate, thereby prolonging the life of the coatings. This multifaceted utility underscores the importance of HEAs in modern engineering and technology development, highlighting the need for a thorough understanding and optimization of their thermal expansion characteristics to meet diverse industrial requirements.

A comparative result of the thermal expansion coefficient of the base HEA and, Nb10, Ta10, and Ti0-HEA's are shown in Table 4.

**Table 4:** TEC of HfNbTaTiZr-HEA and Nb10 Ta10 and Ti0 compositions.

Composition	Thermal Expansion Coefficient (/K)	
	This study	Ref.
HfNbTaTiZr	$1.96\times 10^{-5}$	$2.11\times 10^{-5}$ [45]
Nb10	$6.56\times 10^{-6}$	
Ta10	$8.91\times 10^{-6}$	
Ti0	$6.98\times 10^{-6}$	

The TEC value of the optimized all HEA is lower than the equimolar HfNbTaTiZr-HEA. When the proportion of Hafnium (Hf) increases in the optimized HEA structures, there is a tendency for the linear thermal expansion coefficients to decrease, owing to Hf's inherently lower coefficients in the range of 10^−6^ [40]. However, the results signify that those HEA alloys could potentially be used as a thermal shielding material.

## 4 Fracture evolution

**Fig. 9:**
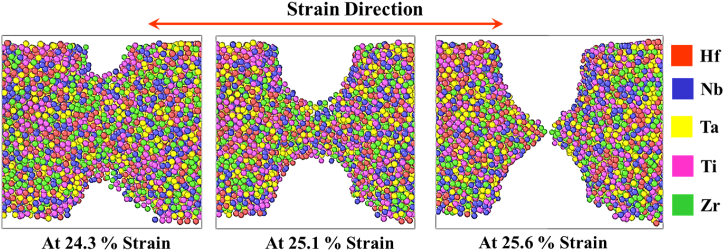
Fracture evolution of Ta10.

The fracture evolution of the Ta10 sample is illustrated in Fig. 9. It is noted that the nature of fracture formation was the same for all the samples. Instantly, after reaching the ultimate tensile stress the atoms slipped to a surface, which results in a 45° angle along the direction of load. At this stage, irreversible deformation of the sample occurs. Due to the breakage of the intermetallic bonds between atoms, a decreased level of force has the potential to cause significant damage to the sample. Once a considerable number of bonds have been broken, the pattern of the fracture becomes noticeable. The snapshots in Fig. 9 can easily describe the fracture of the sample. It was also observed that cup and cone-type fractures propagated during strain.

**Fig. 10:**
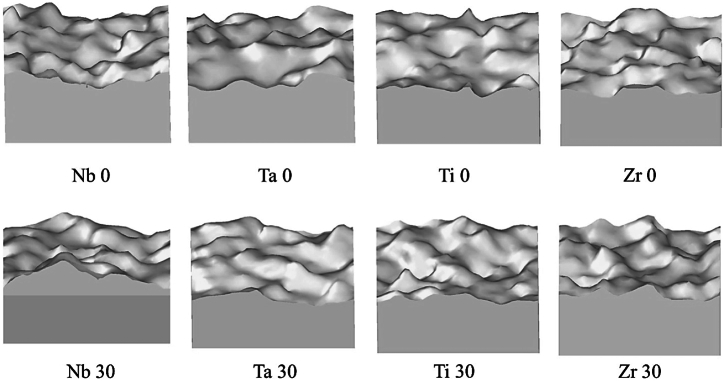
The surface mesh of the fractured samples constructed by Gaussian density method.

The cup and cone morphology can be examined further with the help of surface mesh which is shown in Fig. 10Error! Reference source not found. The surface mesh has been created for the 0 % and 30 % samples for every constituent with the aid of the Gaussian density method [46]. Smooth shading has been selected for rendering the surface. Based on the information presented in Fig. 10Error! Reference source not found., it is possible to predict that the failure of the alloys occurred at the location where the maximum shear stress was generated. This result suggests that these alloys are more susceptible to failure under shear stress rather than tension.

## 5 Conclusion

The variation of properties of HfNbTaTiZr High Entropy Alloy due to changes in alloying composition and temperature are thoroughly studied in this study. The tensile strength, modulus of elasticity, deviation of elongation due to composition change, the most suitable composition of HfNbTaTiZr High Entropy Alloy to be used in high-temperature applications, Fracture Surface of the alloy are thoroughly discussed. The major conclusions drawn from this study are:

• Among the five elements of HfNbTaTiZr High Entropy Alloy Nb10, and Ta10 have a better combination of strength, and Modulus of elasticity. Among them, Ta10 has a superior elongation value of 25.5 % whereas the base alloy had 17 % elongation in tension.
• The high-temperature behavior of the optimized three compositions namely, Nb10, Ta10, and Ti0 showed a similar effect however, Ti0 showed a slightly better performance than the other two combinations.
• The Thermal Expansion Coefficient of the optimized HEA compositions was found smaller than HfNbTaTiZr-HEA, which could be a potential material for high-temperature applications. Whereas, Nb10 showed the least value of $6.56\times 10^{-6}$ K^−1^ among the three compositions whereas Ta 10 has the highest value of $8.91\times 10^{-6}$ K^−1^
• The evaluation of the fracture surface of the alloy shows a cup and cone-type ductile fracture appearance.

For further consideration, multiscale modeling such as Finite Element Analysis (FEA) along with Molecular dynamics can help to assess macroscopic properties. Machine learning prediction of optimized structures can improve accuracy.

## Data availability statement

Data will be made available on request.

## CRediT authorship contribution statement

**Wasif Abu Dujana:** Writing – original draft, Data curation, Conceptualization. **Sazzad Ahmad:** Writing – review & editing, Supervision, Project administration, Funding acquisition, Conceptualization. **Md. Nazmul Haque Noman:** Writing – review & editing, Validation, Software, Methodology, Investigation. **Mohammad Humaun Kabir:** Writing – review & editing, Formal analysis, Conceptualization.

## Declaration of competing interest

The authors declare that they have no known competing financial interests or personal relationships that could have appeared to influence the work reported in this paper.
